# Supplementary material for: Saccadic inhibition interrupts ongoing oculomotor activity to enable the rapid deployment of alternate movement plans
Source: Sci Rep. 2018 Sep 21;8:14163. doi: 10.1038/s41598-018-32224-5 (PMC6155112; doi:10.1038/s41598-018-32224-5)
Supplement: Supplementary file 1 — Supplementary Information [file 41598_2018_32224_MOESM1_ESM.pdf]

Supplementary information

Saccadic inhibition interrupts ongoing oculomotor activity to enable the rapid deployment of alternate movement plans

Emilio Salinas and Terrence R. Stanford

Department of Neurobiology and Anatomy, Wake Forest School of Medicine, 1 Medical Center Blvd., Winston-Salem, NC 27157-1010, USA

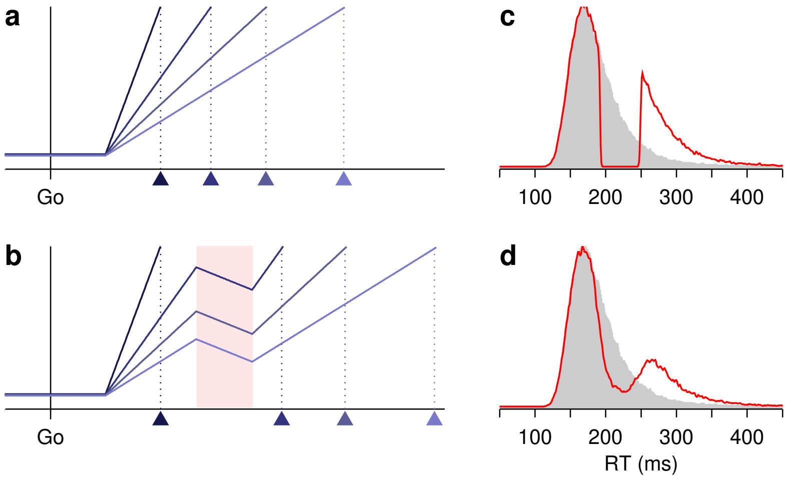

Supplementary Figure 1. Interruptions in motor planning during which activity is suppressed. Simulation parameters were exactly the same as in Fig. 1, except that, during the pause in the rise-to-threshold process, activity decreased at a constant rate. The total drop in activity was equal to 15% of the excursion to threshold. All panels are in the same format as in Fig. 1. **a**, Four example motor plans with no interruption. **b**, Four example motor plans that are briefly interrupted. The oculomotor activity decreases at a constant rate during the interruption period (red shade). **c**, Simulated RT distributions for motor plans that rise to threshold uninterrupted (gray shade) or that are suppressed (red line) for 36 ms (between 192 and 228 ms after the go signal) but are otherwise identical. Note that the void in the latter distribution is longer than 36 ms. **d**, As in **c**, except that the probability of interruption for any given trial was equal to 0.7 (rather than 1), and the onset and offset of the interruption interval varied normally with a SD equal to 8 ms (instead of 0). Note the smooth dip in the distribution. Compared to interruptions in which the firing rate remains constant, activity suppression produces a wider dip, everything else being equal.

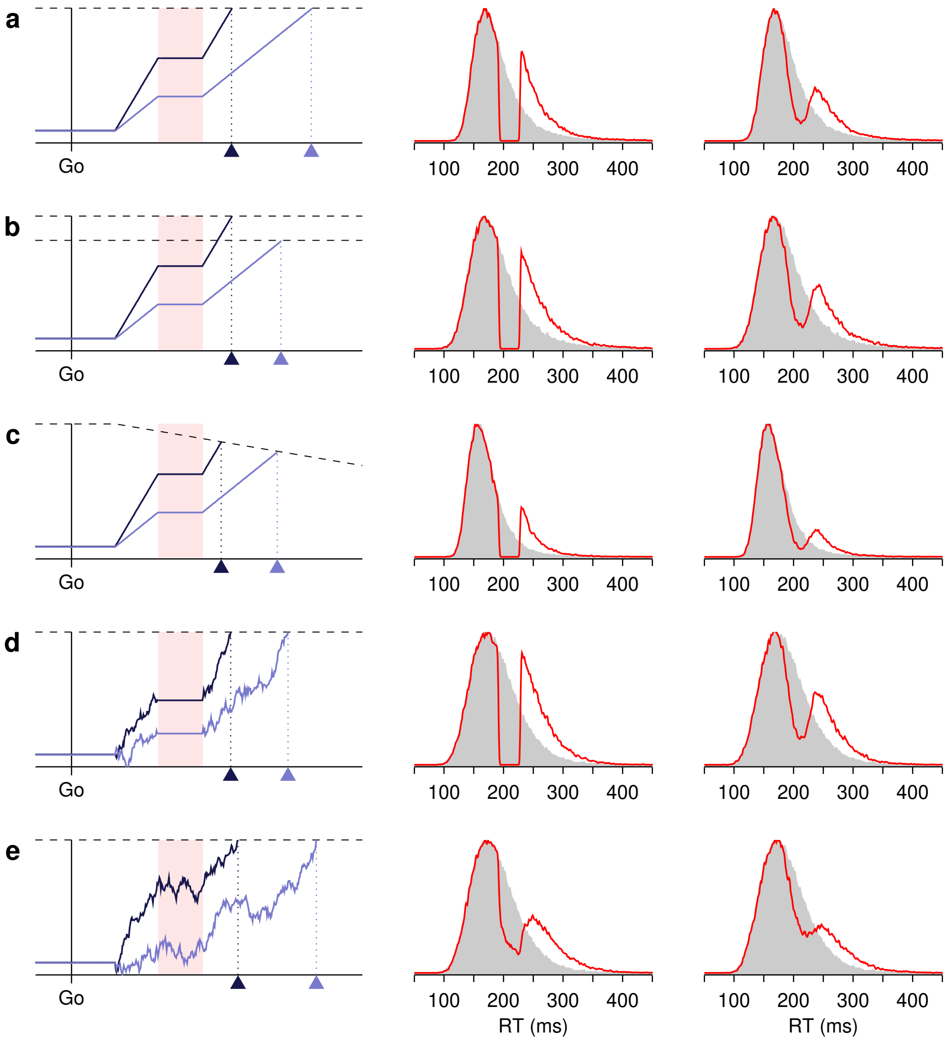

Supplementary Figure 2. Interruptions in motor planning within alternative saccade generation models. Each row shows simulation results for a different variant of the rise-to-threshold mechanism. For each variant, two examples of interrupted trials are shown (left column), along with RT distributions for uninterrupted trials (gray shades), for trials in which the interruption had no variability (middle column, red traces), and for trials in which the interruption varied across trials (right column, red traces). For comparison, the top row shows the same results as in Fig. 1, with the data in all the other rows generated with identical interruption parameters (probability of occurrence, mean onset and offset, and variability). **a**, Results for the linear rise to threshold (same as in Fig. 1). Without variability (middle plot), interruptions lasted exactly 36 ms, occurring between 192 and 228 ms after the go signal. With variability (right plot), the probability of interruption was equal to 0.7, and the onset and offset of the pause varied normally with a SD of 8 ms around their mean values (192 and 228 ms). **b**, As in **a**, except with a variable threshold. The saccade threshold fluctuated normally across trials with a SD equal to 150 AU (i.e., 15% of its mean value). This widened slightly all the RT distributions. The same effect is produced by variations in baseline activity. **c**, As in **a**, except with a collapsing threshold. Starting at the same time as the build-up of activity, the threshold decreased at a rate of 1 AU/ms, so it would take 1 s for it to decrease all the way to baseline level. The effect of this manipulation is to slightly narrow the RT distributions and

shift them to the left. **d**, Rise to threshold by means of drift and diffusion. The net build-up rate (i.e., the drift) was the same for all trials (7.35 AU/ms), but at each time point along the trial ($\Delta t =$ 1 ms), a Gaussian random sample (SD equal to 25 AU) was added to the ongoing firing rate level. This diffusion noise fully accounted for the fluctuations in RT across trials. The drift and diffusion values were chosed so that, in the uninterrupted case (gray shades), the mean and SD of the RTs was the same as in **a**. In this case, during the interruption interval the firing rate level was kept perfectly constant (i.e., both the drift and the diffusion were zero). **e**, As in **d**, except that during the interruption, the drift was zero but the diffusion noise was still present. This, in effect, added variability to the interruption duration. Note that the interruption in motor planning manifests similarly in all cases, even though the dynamics of the build-up process vary drastically.

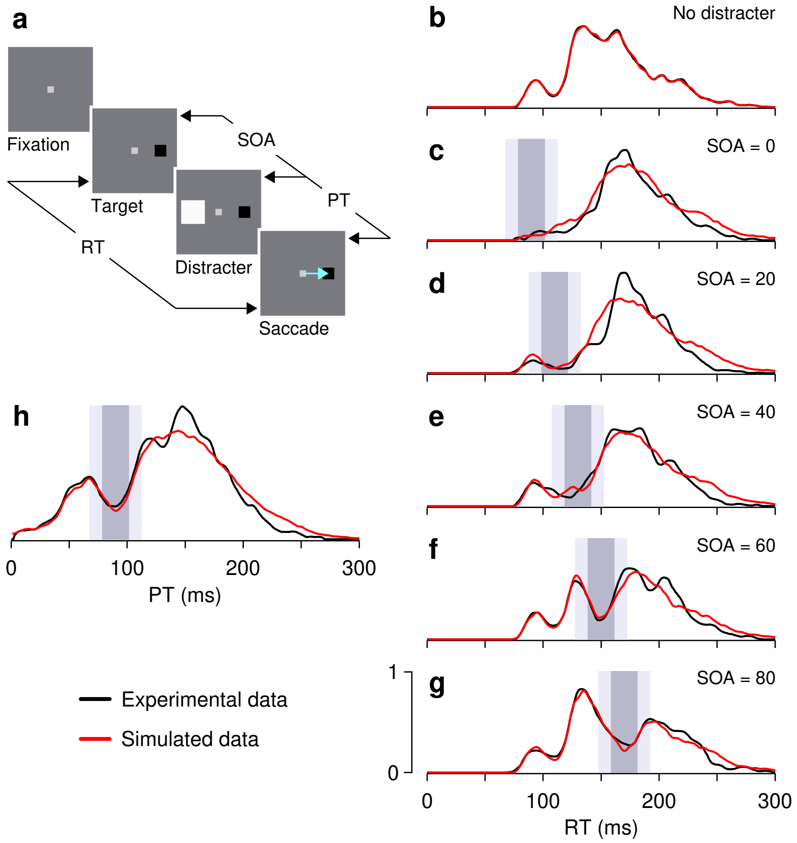

Supplementary Figure 3. Additional comparison between model interruptions in motor planning and experimentally measured dips in RT distributions. Same format as in Fig. 2, except with data from observer 3 from the experiment of Bompas and Sumner[47] (instead of observer 1). Modeling results are based on the same analyses and fitting procedures used for Fig. 2 (Methods). **a**, Schematic of the experimental paradigm. **b–g**, Measured RT distributions[47] (black traces, 1200 trials per trace) along with our model results (red traces). In the rise-to-threshold model, the mean interruption occurred from 79 to 101 ms after distracter onset (dark gray shades), with the onset and offset times varying across trials (light gray shades show 1 SD, equal to 11 ms, in each direction). The interruption probability, p_I, was equal to 1 in distracter-present trials. **h**, PT distributions for the simulated (red trace) and experimental data (black traces). All experimental data were redrawn from Bompas and Sumner[47].
